# Accuracy of non-invasive measurement of cardiac output using electrical cardiometry in preterm infants during the transitional period: A comparison with transthoracic Doppler echocardiography

**DOI:** 10.1007/s00431-025-06132-6

**Published:** 2025-04-15

**Authors:** Silvia Martini, Mariarosaria Annunziata, Jacopo Lenzi, Samir Gupta, Topun Austin, Luigi Corvaglia

**Affiliations:** 1 Neonatal Intensive Care Unit, IRCCS Azienda Ospedaliero, Universitaria Di Bologna, Bologna, Italy; 2 Department of Medical and Surgical Sciences, University of Bologna, Bologna, Italy; 3 Department of Biomedical and Neuromotor Sciences, University of Bologna, Bologna, Italy; 4 Division of Neonatology, Department of Pediatrics, Sidra Medicine, Doha, Qatar; 5 Neonatal Intensive Care Unit, The Rosie Hospital, Cambridge University Hospitals, Cambridge, UK

**Keywords:** Preterm infants, Electrical velocimetry, Echocardiography, Non-invasive cardiac output monitoring, Accuracy, Patent Ductus arteriosus

## Abstract

This prospective observational study aimed to assess the agreement of cardiac output measurements obtained with transthoracic echocardiography (CO_ECHO_) and electrical velocimetry (CO_EV_) and the impact of relevant variables on CO_EV_ accuracy in preterm infants during the transitional period. Simultaneous measurements of CO_EV_ and CO_ECHO_ were performed in preterm infants < 32 weeks’ gestation and/or < 1500 g during the first 72 h of life. Bland–Altman analysis was performed and bias and mean percentage error (MPE) were calculated. The impact of a hemodynamically significant duct (hsPDA), ongoing cardiovascular drugs and ventilatory support was also assessed using a generalized least squares random-effects model. A total of 170 pairs of CO_EV_-CO_ECHO_ measurements were obtained from 65 preterm neonates. Mean bias was 9.7 ml/kg/min (95%CI 1.3–18.2) on day 1, 8.3 ml/kg/min (95%CI 0.3–16.4) on day 2, and 10.6 ml/kg/min (95%CI 4.5–16.6) on day 3 of life. The corresponding MPE was 7.2% (95%CI 4.8–10.6%), 7.5% (95%CI 4.7–12.8%) and 7.0% (95%CI 5.4–9.1%), respectively. A CO_EV_ overestimation was observed in the presence of hsPDA (mean bias = 17.0 ml/kg/min, 95%CI 7.1–30.8, *p* = 0.003) and during dobutamine treatment (mean bias = 12.5 ml/kg/min, 95%CI 1.5–22.4, *p* = 0.018). No significant differences were observed according to dopamine administration and respiratory support modality. *Conclusion*: Although a slight overestimation may occur during inotropic treatments and in the presence of a hsPDA, this study shows an acceptable accuracy and precision of CO_EV_ in preterm infants during postnatal transition, thus supporting the role for EV monitoring in this critical phase.

**Table:** 

**What is Known:** *• Electrical velocimetry allows a continuous and non-invasive monitoring of cardiac output (CO) in the neonatal population.* *• Available data comparing the accuracy of electrical velocimetry against transthoracic echocardiography for CO assessment in preterm infants are still controversial.* **What is New:** *• The present data report a satisfactory accuracy of electrical velocimetry for CO assessment, with low bias and mean percentage error when compared to echocardiographic CO measurements.* *• Inotropic treatment with dobutamine and a hemodynamically significant duct may be associated with a slight but significant overestimation of CO measurements by electrical velocimetry.*

**Supplementary Information:**

The online version contains supplementary material available at 10.1007/s00431-025-06132-6.

## Introduction

Due to their immaturity, preterm infants are at high hemodynamic risk, especially during postnatal transition. Cardiac output (CO) evaluation can add valuable information to standard clinical and vital sign assessments to detect low-flow states and undertake targeted treatments.

Several techniques, including transthoracic echocardiography (TTE) and electrical velocimetry (EV), can be used for non-invasive CO assessment. Although TTE is considered the gold-standard technique in neonates, it only allows intermittent evaluations and is prone to substantial intra- and inter-operator variability [1]. By analysing the pulsatile fluctuations in thoracic electrical bioimpedance in relation to peak aortic blood flow acceleration, EV enables a continuous CO monitoring. Nevertheless, data comparing EV accuracy against TTE for CO assessment in preterm infants are controversial [2–7].

We aimed to assess the agreement between TTE and EV for CO estimation and the impact of relevant clinical variables on EV accuracy in preterm infants during the transitional period.

## Methods

This is a sub-analysis of the NEO-ICM study, including prospectively collected data from infants < 32 weeks’ gestation and/or < 1500 g admitted to the Neonatal Intensive Care Unit of IRCCS AOUBO (Bologna, Italy) between March 2018 and August 2021. Major congenital malformations, congenital heart disease and conditions with a potential influence on the study parameters, such as anaemia (haematocrit < 30%) or persistent pulmonary hypertension requiring inhaled nitric oxide, were exclusion criteria. The study was approved by the Ethics Committee of S. Orsola-Malpighi Hospital, Bologna, Italy (328/2017/O/Oss) and was conducted in conformity with the Helsinki Declaration. Written informed consent was obtained from the infants’ parents.

Over the first 72 h of life, the infants underwent continuous EV monitoring of CO (CO_EV_) using an ICON ® device (Osypka Medical Inc., Berlin, Germany) with beat-to-beat sampling frequency. Neonatal sensors (Cardiotronic™, Osypka Medical Inc., Berlin, Germany) were placed as per manufacturer’s recommendations. During this period, daily echocardiographic scans were performed using an ultrasound scanner CX50 (Philips Healthcare, Amsterdam, The Netherlands) with a linear 12-MHz probe to evaluate left cardiac output (CO_ECHO_) and the ductal status. CO_ECHO_ was calculated according to the formula [(left ventricular outflow [LVO] × velocity time integral [VTI]) × (heart rate) × (LVO cross-sectional area)]. LVO diameter was measured from the parasternal long axis view using the leading-edge technique between the hinges of the aortic valve. VTI was estimated from an apical five-chamber view with pulse-waved Doppler on the LVO tract, applying the insonation angle correction (< 30°) as appropriate. CO_ECHO_ values were averaged over 5 cycles and used for Bland–Altman analysis. CO_ECHO_ measurements were performed by a single trained operator (S.M.), blind to EV data at the time of the scan. At each scan, the ductal status was also assessed and classified as: hemodynamically significant (hsPDA) in the presence of a pulsatile shunt pattern (end-diastolic to peak-systolic velocity ratio ≥ 0.5) and left-atrium-to-aortic-root (LA:Ao) ratio ≥ 1.5 and/or evidence of absent/reversed end-diastolic flow in the descending aorta (DAo) and/or in the anterior cerebral artery (ACA) [8]; restrictive in the presence of a restrictive shunt pattern (end-diastolic to peak-systolic velocity ratio < 0.5), LA:Ao ratio < 1.5 and normal end-diastolic flow in DAo/ACA; closed if no duct was evident.

After the recording, EV traces were reviewed for potential artifacts; signal goodness was assessed to improve artifact detection [6]. CO_EV_ values simultaneous to CO_ECHO_ assessments and averaged over 30 s were used for the Bland–Altman analysis. Both CO_EV_ and CO_ECHO_ were indexed for the infants’ weight. Clinical variables potentially influencing CO estimation were also reviewed for each day of assessment and included in the analysis.

### Statistical analysis

The agreement between CO_EV_ and CO_ECHO_ was assessed using the Bland–Altman plot, with CO_ECHO_ as reference. The 95% limits of agreement (LOA) were defined as the mean difference between CO_EV_ and CO_ECHO_ ± 1.96 times the standard deviation of the differences. To handle repeated measures, the analysis was performed separately on each day of life. The formula $\left|({CO}_{EV}-{CO}_{ECHO})/{CO}_{ECHO}\right|\times 100$ was used to calculate the mean percent error (MPE). The 95% confidence interval (CI) of mean error was estimated using the bootstrap method. Differences in agreement according to the ductal status (hsPDA vs. restrictive/closed duct), ongoing cardiovascular drugs (dobutamine and dopamine) and respiratory support (non-invasive, conventional and high-frequency oscillatory ventilation) were investigated using a generalized least-squares random-effects model, with the absolute delta between CO_ECHO_ and CO_EV_ as the dependent variable. Data were analysed using Stata 18 (StataCorp. 2023. Stata Statistical Software: Release 18. College Station, TX:StataCorp LLC).

## Results

**Fig. 1:**
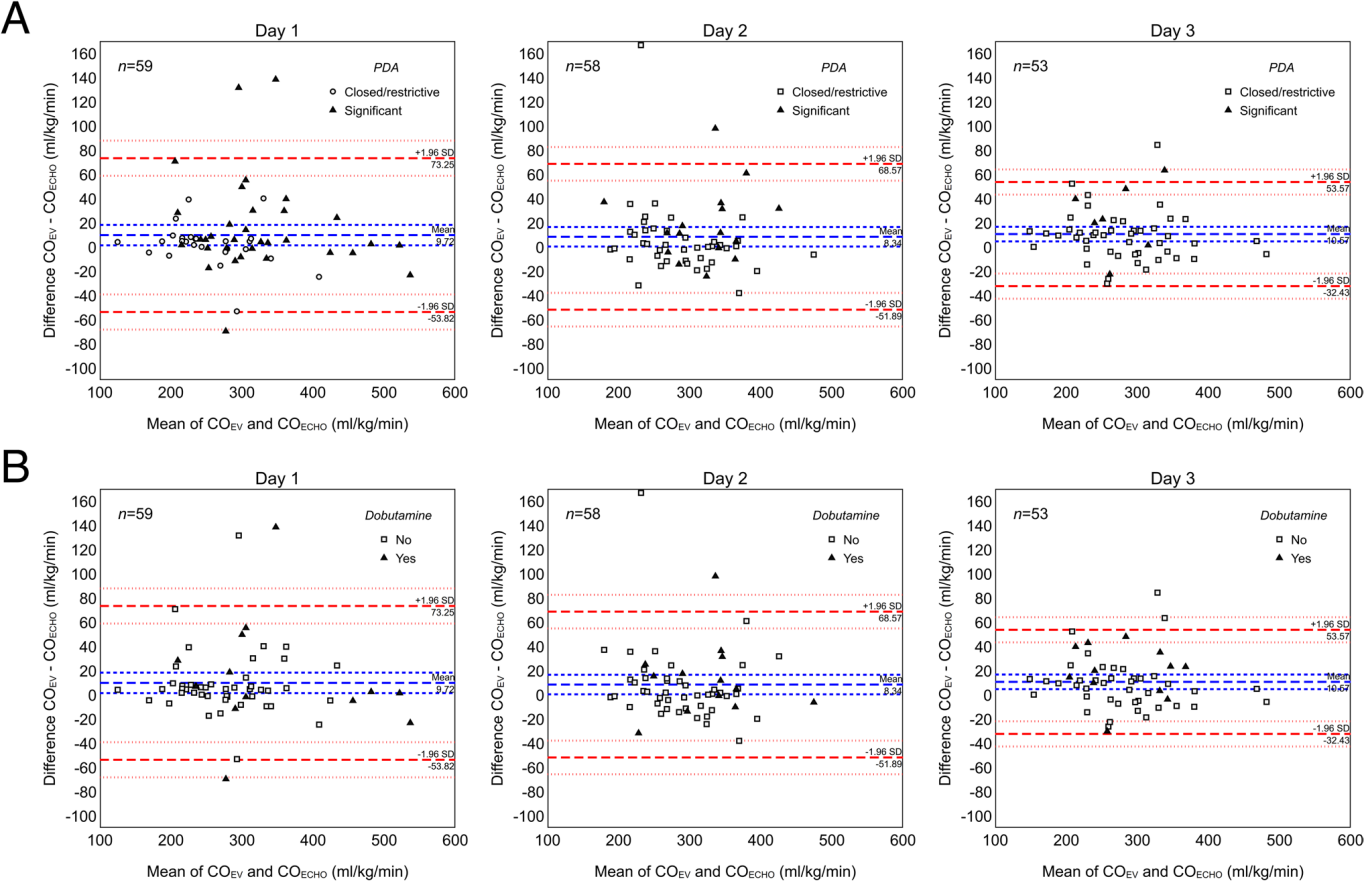
Bland–Altman plot of cardiac output measured with electrical velocimetry (CO_EV_) versus echocardiography (CO_ECHO_) on day 1, 2 and 3 of life according to the presence of a haemodynamically significant patent ductus arteriosus (PDA) (panel A) and to dobutamine administration (panel B). Red-dashed lines indicate the upper and lower limit of agreement (mean CO_EV_-CO_ECHO_ difference ± 1.96 * standard deviation [SD]). Short-dashed lines indicate the 95% confidence interval for the mean difference

A total of 170 pairs of CO_EV_-CO_ECHO_ measurements (59 on day 1, 58 on day 2, 53 on day 3) were obtained from 65 preterm neonates, whose clinical and hemodynamic characteristics are detailed in Table 1. The mean difference between CO_EV_ and CO_ECHO_ was 9.7 ml/kg/min (95%CI 1.3–18.2) on day 1, 8.3 ml/kg/min (95%CI 0.3–16.4) on day 2, and 10.6 ml/kg/min (95%CI 4.5–16.6) on day 3. The corresponding MPE was 7.2% (95%CI 4.8–10.6%) on day 1, 7.5% (95%CI 4.7–12.8%) on day 2 and 7.0% (95%CI 5.4–9.1%) on day 3. As shown in Fig. 1, there was no evidence of proportional bias. LOA were –53.8 to 73.3 ml/kg/min on day 1, –51.9 to 68.6 ml/kg/min on day 2, and –32.4 to 53.6 ml/kg/min on day 3. Five out of 170 measurements showed a mean CO_EV_-CO_ECHO_ difference above the 95%CI for upper LOA; clinical data associated with these measurements are available as Supplemental Material.

**Table 1:** Clinical characteristics of the study infants at baseline and during the transitional period

**Baseline characteristics (n = 65)**			
Gestational age, mean (standard deviation, SD)	29.4 (2.6)		
Birth weight, mean (SD)	1190 (351)		
Sex (males), n (%)	35 (53.8)		
Small for gestational age, n (%)	13 (20)		
Antenatal steroids (complete course), n (%)	48 (73.8)		
Chorioamnionitis, n (%)	9 (13.8)		
Type of delivery (C-section), n (%)	56 (86.2)		
Cord lactate (mmol/L), mean (SD)	3.2 (1.5)		
CRIB-II score, mean (SD)	7 (4)		
Apgar score, mean (SD)	8 (1)		
**Monitoring period (days of life)**	**Day 1**	**Day 2**	**Day 3**
Weight (g), mean (SD)	1176 (351)	1131 (344)	1078 (339)
Age at evaluation (hours), mean (SD)	12.5 (5)	37 (4.5)	63 (5.6)
CO_EV_ (ml/kg/day), mean (SD) CO_ECHO_ (ml/kg/day), mean (SD)	287 (85) 297 (84)	292 (65) 301 (62)	275 (70) 286 (68)
Cardiac shunts, n (%)			
Hemodynamically significant PDA	36 (55.4)	18 (27.7)	13 (20)
Patent foramen ovale	63 (96.9)	63 (96.9)	62 (95.4)
Blood pressure (mmHg), mean (SD)			
Systolic	47 (6)	51 (7)	54 (6)
Mean	34 (5)	39 (6)	40 (5)
Diastolic	26 (5)	30 (6)	30 (5)
Ongoing cardiovascular drugs, n (%)			
Dobutamine^a^	16 (24.6)	13 (20)	12 (18.4)
Dopamine^b^	14 (21.5)	7 (10.8)	6 (9.2)
Surfactant administration, n (%)	35 (53.8)	38 (58.5)	38 (58.5)
Respiratory support, n (%)			
High-frequency ventilation	3 (4.6)	3 (4.6)	2 (3.1)
Conventional mechanical ventilation	14 (21.6)	13 (20)	12 (18.4)
nCPAP or Bilevel	45 (69.2)	42 (64.6)	34 (52.3)
High-flow nasal cannulas	0 (0)	0 (0)	7 (10.8)
Self-ventilating in air	3 (4.6)	7 (10.8)	10 (15.4)
Mean airway pressure^c^ (mmHg), mean (SD)	8.5 (1.4)	8.6 (1.5)	9.1 (1.5)
Haemoglobin (g/dl), mean (SD)	15.9 (2.1)	15.4 (2.6)	15.3 (2.9)
Lactate (mmol/L), mean (SD)	2.5 (1.4)	2.2 (1.2)	1.8 (0.7)
pH, mean (SD)	7.33 (0.06)	7.36 (0.04)	7.35 (0.04)

^a^ *Clinical indication: evidence of reduced cardiac contractility at echocardiography, either with or without significant hypotension (maximum dosage: 5 mcg/kg/min)*

^b^ *Clinical indication: hypotension refractory to the use of dobutamine or associated with decreased urine output (maximum dosage: 5 mcg/kg/min)*

^c^ *Invasively ventilated infants only*

In the presence of hsPDA (*n* = 56), CO_EV_ was slightly but systematically higher than CO_ECHO_ (mean bias = 17.0 mg/kg/min, 95%CI 7.1–30.8, *p* = 0.003) compared to measurements associated with a restrictive or closed duct. A similar result was observed during dobutamine administration (*n* = 39, mean bias = 12.5 mg/kg/min, 95%CI 1.5–22.4, *p* = 0.018). No significant differences were found according to dopamine administration (*n* = 22, *p* = 0.252) and invasive ventilation, both conventional (*n* = 31, *p* = 0.948) and oscillatory (*n* = 8, *p* = 0.812).

## Discussion

The present study investigated the agreement between CO estimation by EV and TTE in preterm infants during postnatal transition, reporting an overall good agreement between the two techniques and satisfactory EV accuracy.

As described in a recent systematic review [9], EV proved better than other thoracic electrical biosensing technologies in terms of agreement with CO_ECHO_ in the neonatal population; nevertheless, the reported bias, either negative or positive, varies significantly among the available studies, half of which reported a MPE > 30% [2–6].

EV would represent a useful tool for non-invasive CO monitoring in preterm infants, who are prone to significant hemodynamic instability. To date, however, the evaluation of the agreement between CO_EV_ and CO_ECHO_ in this population has yielded variable results. While a relatively good consistency (i.e., low bias, narrow LOA) and a MPE < 30% was reported by several studies [2, 10, 11], others described a poorer agreement and accuracy [3, 4, 6]. Methodological factors, such as the inclusion of infants with different baseline characteristics (e.g., gestational and postnatal age, body size) or the occurrence of technological advancements (i.e., more precise algorithms, introduction of neonatal sensors) over the period during which these studies were performed, may underlie these heterogeneous findings.

Since the need for arterial catheterization limits the applicability of transpulmonary thermodilution for CO assessment in neonates, TTE is considered the clinical gold-standard for non-invasive CO estimation in this population. Nevertheless, echocardiographic CO measurements are not exempt from a significant inter- and intra-operator variability, and a MPE around 30% compared to thermodilution-derived measurements has been reported [1]. Hence, TTE may not represent the best reference method for CO_EV_ validation, and an increase of MPE threshold up to 45% has been suggested to compensate for CO_ECHO_ variability.

According to our results, hsPDA was associated with a significant CO_EV_ overestimation compared to CO_ECHO_, consistently with previous data [12]. A significant hsPDA impact on both CO_EV_ and CO_ECHO_, although with a negative bias, was also reported by other studies [3, 10]. The interference of transductal shunt on volumetric changes and on the aortic alignment of erythrocytes during the cardiac cycle may underlie this finding.

To our knowledge, this is the first study investigating the influence of cardiovascular drugs on CO_EV_ accuracy. While no significant effect was observed with vasopressor agents, such as dopamine, a slight but significant CO_EV_ overestimation occurred during inotropic treatment with dobutamine; however, further validation is required to confirm this result and hypothesize potential underlying mechanisms.

In the present study, neither conventional nor high-flow oscillatory ventilation were associated with a significant proportional bias. Our results are in line with previous data reporting no significant effects of ventilatory modalities [5, 10]. Conversely, Hassan et al. described a lower bias in association with HFOV [3], whereas opposite evidence of higher bias and PE was reported by two studies [2, 4]. These variable findings may be ascribable to the different characteristics of infants requiring HFOV (gestational age, hemodynamic instability, hsPDA) and to the noticeably low number of HFOV measurements.

In the presence of systemic hypoperfusion, EV may play a potentially relevant role for CO monitoring; however, the limited number of infants with a left ventricular output < 150 ml/kg/min, which defines a low-flow state, limits the generalizability of the present results to this condition, which therefore requires targeted investigations.

Our data overall support the role for CO_EV_ monitoring in preterm infants during postnatal transition. The use of neonatal sensors, avoidance of inter-operator bias for CO_ECHO_ and the relatively homogeneous characteristics of the study population may have contributed to the low bias and MPE. However, a slight CO_EV_ overestimation was observed in association with hsPDA and during dobutamine treatment, highlighting the importance of complementary echocardiographic assessments for clinical decision-making, especially in these conditions. Large and well-designed studies allowing to adequately analyse population subsets (e.g., different gestational and weight ranges) and the impact of clinical and hemodynamic factors are needed to better define CO_EV_ accuracy and precision.

## Supplementary Information

Below is the link to the electronic supplementary material.

Supplementary file1 (DOCX 16 KB)

## Data Availability

Datasets are available from the corresponding author upon reasonable request.

## Abbreviations

ACA: Anterior cerebral artery
CI: Confidence interval
CO: Cardiac output
CO_ECHO_: Cardiac output measured by echocardiography
CO_EV_: Cardiac output measured by electrical velocimetry
DAo: Descending aorta
EV: Electrical velocimetry
hsPDA: Hemodynamically significant patent ductus arteriosus
LA:AO ratio: Left-atrium-to-aortic-root ratio
LOA: Limit of agreement
LVO: Left ventricular outflow
MPE: Mean percentage error
TTE: Transthoracic echocardiography
VTI: Velocity time integral

## Code availability

N/A.
