# Supplementary material for: Accuracy of non-invasive measurement of cardiac output using electrical cardiometry in preterm infants during the transitional period: A comparison with transthoracic Doppler echocardiography
Source: Eur J Pediatr. 2025 Apr 15;184(5):299. doi: 10.1007/s00431-025-06132-6 (PMC11996928; doi:10.1007/s00431-025-06132-6)
Supplement: Supplementary file 1 — Supplementary file1 (DOCX 16 KB) [file 431_2025_6132_MOESM1_ESM.docx]

***Supplemental Material***

*Clinical characteristics associated with outlier measurements.*

|  | **CO_EV_-CO_ECHO_ Δ (ml)** | **GA (weeks)** | **BW**  **(g)** | **DOL** | **Ductal status** | **Respiratory support (FiO_2_)** | **Cardiovascular drugs (mcg/kg/min)** |
| --- | --- | --- | --- | --- | --- | --- | --- |
| 1 | 131.48 | 31 | 1080 (SGA) | 1 | hsPDA | nCPAP (0.21) | None |
| 2 | 166.67 | 31 | 1080 (SGA) | 2 | Closed | nCPAP (0.21) | None |
| 3 | 84.26 | 31 | 1080 (SGA) | 3 | Closed | SVIA | None |
| 4 | 97.81 | 27 | 961 | 1 | hsPDA | CMV (0.21) | Dopamine (3), dobutamine (5) |
| 5 | 138.33 | 29 | 1200 | 1 | hsPDA | CMV (0.21) | Dopamine (5), dobutamine (5) |

*Abbreviations (in alphabetical order): BW, birth weight; CO, cardiac output; CMV, conventional mechanical ventilation; DOL, day of life; GA, gestational age; hsPDA, haemodynamically significant patent ductus arteriosus; nCPAP, nasal continuous positive airway pressure; SGA, small for gestational age; SVIA, self-ventilating in air.*
